# Pre-SAFEx: protocol for a single-centre, randomised, unblinded feasibility trial of Simultaneous Application of Flow at Extubation (SAFEx) in patients requiring intubation and ventilation for the management of acute respiratory failure

**DOI:** 10.1186/s40814-026-01817-7

**Published:** 2026-04-08

**Authors:** Duncan G. Thomson, Glasiele C. Alcala, Malcolm J. Watson, Malcolm A. B. Sim

**Affiliations:** 1 School of Medicine, Dentistry and Nursing, College of Medical, Veterinary and Life Sciences, University of Glasgow, Glasgow, G12 8QQ Scotland; 2 Department of Critical Care Medicine, Queen Elizabeth University Hospital, 1345 Govan Road, Glasgow, G51 4TF Scotland; 3 University of São Paulo Medical School, São Paulo, Brazil

**Keywords:** Acute respiratory failure, Failed extubation, High-flow nasal therapy, Electrical impedance tomography, Critical care

## Abstract

**Background:**

Failed extubation within critical care is associated with increased morbidity and mortality. Although the role of high-flow nasal therapy (HFNT) has been established post-extubation, it is not clear whether the delay in establishing HFNT after extubation adversely affects lung physiology, resulting in an increased risk of extubation failure. Early administration of HFNT may prevent lung derecruitment and reduce the rate of extubation failure. Electrical impedance tomography (EIT) is a non-invasive, non-ionising imaging method that allows continuous real-time measurement of lung physiology.

**Methods:**

This is a single-centre, unblinded, randomised feasibility trial. Patients who have required intubation and ventilation for the management of acute respiratory failure will be recruited from the critical care unit at Queen Elizabeth University Hospital, Glasgow, Scotland. At the time of their planned extubation, patients will either be randomised to SAFEx extubation (where HFNT is established pre-extubation) or to standard care (where low-flow conventional oxygen therapy is established post-extubation). EIT will be used to compare the lung physiology of these two extubation strategies.

**Discussion:**

This randomised feasibility trial will provide the relevant information to determine whether a multi-centre feasibility study comparing a SAFEx extubation against standard care is viable. It will also determine the feasibility of using EIT as a measure of changes in lung physiology peri-extubation. As extubation failure remains a significant burden for critical care services, with an associated lack of evidence addressing the impact that the oxygen delivery device has on the outcome of extubation in high-risk patients, this study aims to take the first step in addressing this important research question.

**Trial registration:**

ClinicalTrials.Gov. Registration number: NCT05904652. Date of registration: 2023-06-15. https://clinicaltrials.gov/

## Background

Failed extubation is defined as the need for reinstitution of ventilatory support within 24 to 72 h of a planned extubation [1]. The mean rate in one review of interventional and observational studies was 15.7%, but this rate may be greater in high-risk patients [2]. Reintubation is associated with increased mortality [3]. Patients tend to be older, have pneumonia as an underlying diagnosis, have a more severe illness and are more likely to have a more rapid shallow breathing index [3].

High-flow nasal therapy (HFNT) was first described in the 1950s, but systems to deliver both heated and humidified oxygen at gas flows of up to 60 L/min have only been established in the past 15 years [4]. Greater oxygenation is achieved with high-flow devices in respiratory failure patients by better matching the patient’s peak inspiratory flow rate [5, 6]. This results in lower entrainment of and dilution with room air, resulting in more oxygen reaching the alveoli [7]. Another important mechanism is the generation of 3–5 cm H_2_O of positive end-expiratory pressure (PEEP) at flow rates of 30–50 L/min [7]. Humidification aids mucociliary function and secretion clearance and results in high tolerability [6]. There are a wide range of uses for the therapy. Most significantly, a mortality benefit has been demonstrated with its use in acute hypoxaemic respiratory failure [8].

Compared with conventional oxygen therapy, extubation of a patient onto a HFNT results in better oxygenation for the same inspired oxygen concentration after extubation and is associated with better comfort, less desaturation, and a lower reintubation rate [9, 10]. Moreover, in mechanically ventilated patients at high risk of extubation failure, the use of HFNT oxygen with non-invasive ventilation immediately after extubation significantly decreases the risk of reintubation compared with the use of HFNT alone [11]. 

Although the role of HFNT is established post-extubation, it is not clear whether the delay in establishing the therapy after extubation affects lung physiology, leading to an increased risk of reintubation. To characterise peri-extubation changes in lung aeration, two principal imaging modalities have been employed in the literature: lung ultrasound and electrical impedance tomography.

Lung ultrasound (LUS) enables serial assessment of lung aeration when performed using a standardised regional examination and reporting system for 12 regions, allowing aeration to be expressed semi-quantitatively as a score. Four ultrasound patterns reflect progressively worsening aeration loss: normal aeration with A-lines, moderate loss with well-separated B-lines, severe loss with coalescent B-lines, and complete loss with consolidation. An aeration score (0–36) is calculated by summing regional scores based on the worst pattern observed, with increasing scores indicating worsened aeration [12].

In a prospective observational study conducted in two multidisciplinary ICUs, Soummer et al. evaluated whether lung ultrasound score (LUS) could predict post-extubation distress by detecting impaired baseline aeration or derecruitment during a spontaneous breathing trial (SBT). SBTs were performed using a T-piece trial, and patients who passed were extubated. Among 100 mechanically ventilated patients, 86 were extubated after a successful SBT, of whom 34% developed post-extubation distress. In this heterogeneous patient group, higher baseline LUS and greater increases in LUS during the SBT were associated with post-extubation respiratory distress and an increased need for reintubation [13].

Electrical impedance tomography (EIT) is a non-invasive, real-time imaging modality that generates cross-sectional images of pulmonary ventilation. The technique measures changes in lung tissue impedance by applying small alternating electrical currents through skin surface electrodes and recording the resulting voltage differences [14]. The electrical currents emitted by EIT electrodes are inconsistently attenuated in different tissue types. Well-ventilated lung regions demonstrate higher impedance values, whereas poorly ventilated regions exhibit lower values, and unventilated areas demonstrate a minimum impedance value. Although conceptually analogous to computed tomography in generating cross-sectional images, EIT provides continuous, radiation-free bedside monitoring and reflects real-time changes in pulmonary physiology during inspiration and expiration [15].

EIT offers several advantages over lung ultrasound. First, EIT enables continuous data acquisition at up to 50 frames per second, allowing dynamic assessment of pulmonary function before, during and after extubation [16]. In contrast, lung ultrasound provides operator-dependent snapshot measurements. Calculation of a Lung Ultrasound Score (LUS) requires substantial training and expertise, may take up to 15 min to complete and may be susceptible to inter-observer variability [12].

Furthermore, EIT provides both global and regional information on pulmonary physiology down to the level of individual pixels. Functional EIT imaging enables assessment of clinically relevant physiological parameters, including regional ventilation distribution and changes in lung aeration, which are directly pertinent to evaluating lung recruitment peri-extubation [15].

Longhini et al. conducted a multicentre observational study examining changes in lung aeration and ventilation during the first spontaneous breathing trial (SBT) and following extubation in adult ICU patients at increased risk of extubation failure, using electrical impedance tomography (EIT). They found that SBT failure was associated with greater lung derecruitment, worsening oxygenation, and increased ventilation inhomogeneity; however, EIT-derived variables did not significantly differentiate between patients who ultimately succeeded or failed extubation. In this study, patients were extubated following a SBT to a Venturi mask, with CPAP or non-invasive ventilation used as rescue therapy where required [17].

### Study rationale

The patient’s respiratory function should be optimised prior to extubation, as reintubation is associated with increased morbidity and mortality. A strategy that best maintains lung recruitment and aids mucociliary function may reduce failed extubation. Conventional extubation strategies inevitably result in a delay between removal of the endotracheal tube and commencement of either high-flow therapy or low-flow oxygen. Although this period is relatively short, this could lead to aeration changes or derecruitment.

We propose to commence HFNT in patients who are receiving low respiratory support and who, in the opinion of the treating clinician, are deemed ready for extubation. The cuff on the endotracheal tube will remain inflated, and HFNT will commence gradually to achieve a flow of 60 L/minute, allowing humidification of the upper airway. Using EIT, this strategy of extubation with HFNT running will be compared with conventional oxygen therapy being commenced after extubation.

## Methods and analysis

### Study design and setting

The study is a single-centre, unblinded, randomised, feasibility trial. The study protocol was developed in accordance with the Standard Protocol Items: Recommendations for Interventional Trials (SPIRIT) 2013 Statement [18]. It is being carried out at the Queen Elizabeth University Hospital, Glasgow, UK—a tertiary centre and teaching hospital.

### Participant selection

Patients with respiratory failure of  ≥ 48 h who are scheduled for a planned extubation will be screened in critical care.

Written informed consent will be obtained from all patients recruited for the study. Consent will be sought directly from the patient where possible or from their Nearest Relative/Guardian or Welfare Attorney (or nearest ward if no suitable Nearest Relative/Guardian or Welfare Attorney is available) in accordance with the Adults with Incapacity (Scotland) Act 2000 for adults who lack capacity. Trial participants will be informed once they have recovered capacity after extubation that they are free to withdraw from the study at any time, without having to give a reason, and without their current or future care being affected. Written consent will be obtained to allow them to continue in the study.

### Study sample

In planning our feasibility study, we conducted a calculation to determine the required sample size for a future definitive randomised trial of SAFEx management versus standard care using G*Power software [19]. This was based on a 2-group Wilcoxon-Mann-Whitney test. We assumed a logistic distribution for the dependent variable (extubation failure rate) in this future trial, with an effect size of 0.1, an α error probability set at 0.05, and a desired power of 0.80 under a 1:1 randomisation scheme. This calculation suggested a total sample size of 2258 patients (1129 per group) would be required, indicating the need for a multi-centre trial.

We also referred to the work of Teare et al. [20], in which they explore the disagreement that currently exists within the literature over what sample size should be chosen for external pilot trials that inform the design of definitive randomised controlled trials. In their computer-based simulation study, Teare et al. [20] wished to provide recommendations on the required sample size for external pilot randomised controlled trials to enable the estimation of critical parameters that are necessary to inform the design of definitive randomised controlled trials (such as consent rate, event rate and attrition rate) with a reasonable degree of precision whilst avoiding over-recruitment. Their computer-programmed statistical illustration assessed multiple binary outcome success probabilities against a range of sample sizes. They concluded that a target sample size in the range of 60–100 patients per group (120 to 200 patients in total) was adequate.

For this feasibility study, we have chosen a recruitment target of 30 patients over 12 months. This is a pragmatic decision and has been driven by the limited resources we have to carry out this initial pilot. These include limits in staffing (with recruitment being facilitated by a single staff member working part-time), having access to a single Enlight 2100 electrical impedance tomography system and an uncertain local prevalence of eligible patients.

The extubation failure rate in the critical care sub-population with primary acute respiratory failure is not well defined. One review of the literature reports a mean extubation failure rate across all critical care patients of 15.7%, though this is likely to be greater in high-risk groups such as those with acute respiratory failure [2]. With the intervention being considered in this study, we would consider an absolute risk reduction in extubation failure rate of 10% to be clinically significant thus justifying our effect size of 0.1.

While we acknowledge the potential benefits of a larger sample size as recommended by Teare et al. [20], this initial feasibility study serves as a critical first step in understanding the recruitment landscape for our intervention as well as the feasibility of our protocol. The data obtained from this study will inform decision-making regarding the feasibility of a future multi-centre feasibility trial such as the resource considerations to achieve the larger sample size requirements.

### Eligibility criteria

Patients will be eligible to participate if (1) they are aged 18–80 years at the time of recruitment for the study, (2) they have been ventilated for ≥ 48 h with respiratory failure, (3) they are ready for a planned extubation in the opinion of the treating clinician (e.g. the patient is established on pressure support ventilation, FiO_2_ ≤ 0.4, PEEP ≤ 10 cm H_2_0, with a respiratory rate ≤ 20), (4) they have minimal secretions, (5) they are neurologically intact (in the opinion of the treating clinician, the patient is unlikely to fail extubation due to their neurological status), (6) they are cardiovascularly stable (systolic blood pressure ≥ 70 mmHg, heart rate ≤ 150 bpm) and (7) written informed consent has been given.

Exclusion criteria include the following:

#### Medical contraindications

- Severe type II respiratory failure (PaCO_2_ ≥ 12 kPa)
- Severe acidosis ([H^+^] ≥ 80 nmol/L)
- Chronic respiratory disease limiting functional capacity (MRC breathlessness grade IV or V)
- Severe heart failure (NYHA grade III or IV)
- GCS score ≤ 12
- Cardiovascular instability (systolic blood pressure ≤ 69 mmHg or heart rate ≥ 151 bpm)
- Pulmonary embolism
- Life expectancy ≤ 3 months

#### Contraindications to EIT

- Implantable cardiac devices
- Internal neurostimulator
- Unstable spinal fracture or spinal cord injury
- Body mass index > 50 kg/m^2^
- Skin lesions or dressings at the electrode belt site
- Pregnancy or lactation
- Intercostal Chest Drain (at the treating clinician’s discretion)

#### Contraindication to receiving high-flow nasal therapy

- Nasal obstruction
- Previous bleomycin administration
- Base of skull fracture

### Study procedures

**Fig. 1:**
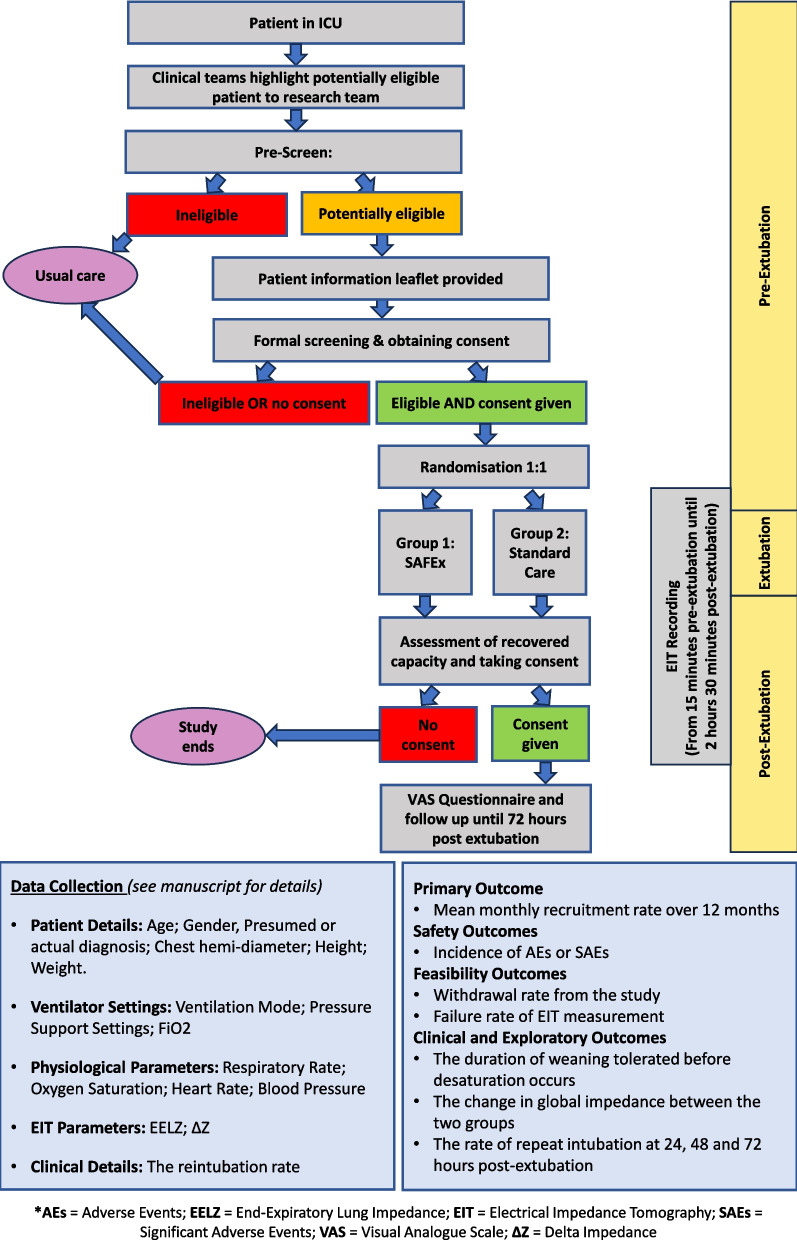
Pre-SAFEx trial flow chart

The Pre-SAFEx study design is summarised in Fig. 1 and described in detail below and in Table 1.

**Table 1:** Participant timeline and schedule of assessments

Study procedure	Visit 1 (Pre-Screen)	Visit 2 (Provision of Patient Information Leaflet)	Visit 3 (Formal screening, answering questions & taking consent)	Visit 4 (Randomisation and undertaking of study)	Visit 5 (Assessment of recovered capacity and taking consent)	Visit 6 (Undertaking Questionnaire)
Assess if patient meets the entry criteria (Pre-Screen)	✓					
Give verbal and written information to • The patient OR • Next of kin (Nearest Relative/Guardian or Welfare Attorney (Scotland)) or nearest ward if no suitable Nearest Relative/Guardian or Welfare Attorney (Scotland) is available		✓				
Assess if patient meets the entry criteria (Formal Screen)			✓			
Patient consented to the study			✓			
Final assessment of eligibility criteria (Cross referenced with medical records)			✓			
**Patient randomised to one of 2 groups:** **Group 1:** • EIT applied and recording commenced 15 min prior to planned extubation • HFNT running at least 10 min prior to extubation. (HFNT will be administered via *Fisher and Paykel Airvo 3* with *Optiflow DUET* nasal cannula) • Start at 10 L/min flow and FiO2 of 0.4 with the cuff of the endotracheal tube inflated—increasing the flow rate gradually to a maximum rate 60L/min or as high as can be tolerated by the patient. At 5 min before extubation, the flow rate should be either at 60 L/min or as high as can be tolerated by the patient. Record the maximum flow that is tolerated by the patient • Endotracheal, infraglottic and supraglottic suctioning prior to extubation • Cuff let down and immediate extubation with simultaneous application of HFNT • 10 min after extubation, FiO2 weaned in a protocolised manner to 0.21 – or as close to 0.21 as possible over 25 min • If successfully weaned onto room air, the flow rate of HFNT is reduced in a protocolised manner over 120 min: 60 min at 60L/min (or highest flow rate tolerated) and 60 min at 30L/min • End of weaning protocol **Group 2:** • EIT applied and recording commenced 15 min prior to planned extubation • Endotracheal, infraglottic and supraglottic suctioning prior to extubation • Cuff let down, patient extubated on to low-flow conventional oxygen with an FiO2 up to 0.4 and then the patient will be weaned at the discretion of their clinician over 2 h and 35 min • End of weaning The electrical impedance tomography/saturation measurements will last in total for 2 h 50 min (15 min before and 2 h 35 min after extubation). After this period, the patient can remain on either HFNT or low-flow oxygen and weaned at the discretion of the treating clinician At an appropriate time after the weaning protocol, patients from both groups will be asked to complete a Visual Analogue Scale Questionnaire exploring the Acceptability and Tolerance of the allocated Oxygen Delivery Device (HFNT or COT) and that of EIT recording. The patient will be followed up at 24, 48 and 72 h post-extubation for any incident of repeat intubation. The study will then conclude at 72 h post-extubation				✓		
**Data collection and time points** ***Baseline data*** Patient details: age; gender’ presumed or actual diagnosis; chest hemi-diameter; height; weight Ventilator settings: ventilation mode; pressure support settings; FiO_2_ Physiological parameters: respiratory rate; oxygen saturation; heart rate; blood pressure ***Pre-extubation*** Parameters within 1 h prior AND 15 min prior to planned extubation (*15 min prior only): Ventilator settings: (as above) Physiological parameters: (as above) EIT parameters*: EELZ; ΔZ ***Parameters within 15 min pre-extubation*** HFNT settings: FiO_2_; flow rate Ventilator settings: (as above) Physiological parameters: (as above) EIT parameters: (as above) ***Extubation*** Type of oral endotracheal tube ***Post-extubation (2 h 35 min)*** **SAFEx group** HFNT settings (as above) **COT group** Details of oxygen delivery device **All groups** EIT parameters (as above) Physiological parameters (as above) ***Post-extubation (0–72 h)*** Reintubation rate: 24, 48 and 72 h Visual Analogue Scale Questionnaire exploring • The Acceptability and Tolerance of the allocated Oxygen Delivery Device (HFNT or COT) • The Acceptability and Tolerance of EIT recording				✓		
**Recovered capacity** Should the patient demonstrate Recovered Capacity, they will be provided with verbal information and a separate Patient Information Leaflet before being invited to give their written consent for ongoing study participation					✓	
**Undertaking Questionnaire** Visual Analogue Scale Questionnaire exploring • The Acceptability and Tolerance of the allocated Oxygen Delivery Device (HFNT or COT) • The Acceptability and Tolerance of EIT recording						✓
Reporting of ***adverse events*** or ***serious adverse events***				✓	✓	✓

#### Randomisation

After providing consent, eligible patients will be randomised in a 1:1 manner using a computer-generated 30 number sequence of ‘1 s’ and ‘2 s’ from https://www.random.org/ between (1) SAFEx treatment and (2) standard care. A clinician not involved in the study will obtain this 30 number sequence and conceal its order within 30 sealed, opaque, numbered envelopes. Participants will be unblinded to the research team following randomisation.

Participants will be randomised to one of two groups:

### Group 1 (SAFEx)

- *Enlight 2100* electrical impedance tomography system will be applied and recording commenced 15 min prior to planned extubation.
- HFNT commenced at least 10 min prior to extubation. (HFNT will be administered via Fisher and Paykel *Airvo 3* with an *Optiflow DUET* nasal cannula.)
- Starting at 10 L/min flow and an FiO_2_ of 0.4 with the cuff of the endotracheal tube inflated, the flow rate will be gradually increased to a maximum rate of 60 L/min or as high as can be tolerated by the participant. Five minutes before extubation, the flow rate should be either 60 L/min or as high as can be tolerated by the participant. The maximum flow that is tolerated by the participant will be recorded.
- Endotracheal, infraglottic and supraglottic suctioning undertaken prior to extubation.
- The endotracheal cuff will then be let down and immediate extubation performed with simultaneous application of HFNT.
- Ten minutes after extubation, FiO_2_ will be weaned in a protocolised manner to 0.21 or as close to 0.21 as possible over 20 min.
- If successfully weaned on to room air (FiO_2_ 0.21), the flow rate of HFNT will be reduced in a protocolised manner over 120 min: 60 min at 60 L/min (or the highest flow rate tolerated) and 60 min at 30 L/min.
- End of weaning protocol

### Group 2 (standard care)

- *Enlight 2100* electrical impedance tomography system will be applied and recording commenced 15 min prior to planned extubation.
- Endotracheal, infraglottic and supraglottic suctioning undertaken prior to extubation.
- The endotracheal cuff will then be let down and immediate extubation performed on to low-flow conventional oxygen with an FiO_2_ up to 0.4
- The participant will then be weaned at the discretion of their clinician over a maximum of 2 h and 30 min to mirror group 1
- End of weaning

An unsuccessful wean is defined as an oxygen saturation of ≤ 93% and a respiratory rate ≥ 31 for longer than 30 s in total within a 5-min interval. Immediately after an unsuccessful wean occurs, the FiO_2_ will be increased by 0.1 to prevent continued desaturation and the study will be stopped.

The study will also be stopped for any of the following reasons: heart rate ≥ 150 bpm at any point; systolic blood pressure ≤ 70 mmHg when recorded at 5-min intervals; SpO_2_ ≤ 88% at any point; or respiratory rate ≥ 40 bpm at any point or there is clinical concern at any point. Clinicians will be able to institute rescue therapy at their discretion. This may include fluid boluses, increasing the flow rate, FiO_2_, non-invasive ventilation or intubation.

The electrical impedance tomography/saturation measurements will last for 2 h 45 min (15 min before and 2 h 30 min after extubation). After this period, the participant can remain on either HFNT or low-flow oxygen and be weaned at the discretion of the treating clinician.

At an appropriate time after the weaning protocol, participants from both groups will be asked to complete a visual analogue scale questionnaire exploring the acceptability and tolerance of the allocated oxygen delivery device (HFNT or COT) and that of EIT recording. The participant will be followed up at 24, 48 and 72 h post-extubation for any incidence of repeat intubation. The study will then be concluded at 72 h post-extubation. 

### Data collection and time points

A participant timeline and schedule of assessments is summarised in Table 1.

### Assessment and measurement

Baseline assessment will include demographic characteristics: age, gender, presumed or actual diagnosis, chest hemi-diameter, height and weight.

The primary feasibility outcome will be the recruitment rate to the study protocol over 12 months with a 1:1 randomisation of patients between SAFEx treatment and standard care. The associated endpoint will be the average rate of recruitment per month over 12 months (with a set upper limit of 30 patients recruited over 12 months, i.e. a recruitment rate of 2.5 patients per month).

The secondary outcomes (including additional measures of feasibility together with clinical and exploratory outcomes) are summarised in Table 2 with their associated outcome measures.

**Table 2:** Secondary outcomes and associated end points in the Pre-SAFEx study

Secondary outcome	Associated endpoint
The feasibility of a SAFEx treatment model peri-extubation using HFNT	The failure rate of the procedure defined as the proportion of patients in whom SAFEx treatment could not be tolerated
The safety of a SAFEx treatment model peri-extubation using HFNT	The incidence of Adverse Events (AEs) and Serious AEs (SAEs) associated with SAFEx treatment
The tolerability of a SAFEx treatment model peri-extubation using HFNT	Patient Visual Analogue Scale scores for questions exploring the tolerability of SAFEx treatment compared with that of standard care. Patients will be asked to rate their experience with the oxygen delivery device they were randomised to for: • Overall comfort • Perceived dyspnoea • Ability to speak • Ability to hear • Ability to clear secretions • Sensation of bloating • Sensation of dry mouth • Sensation of nasal dryness • Fear **and** Withdrawal rate from the study due to inability to tolerate the trial procedure
The rate of completion of the SAFEx weaning protocol	Defined as the percentage of patients who completed the weaning protocol without breaching any of the physiological patient safety criteria **and** The average fraction of inspired oxygen and oxygen flow rate administered in each group before desaturation occurred
The feasibility of impedance measurement in intubated and ventilated patients using the *Enlight 2100* Electrical Impedance Tomography system	The failure rate of the procedure, defined as the proportion of patients in whom impedance data cannot be measured
The safety of impedance measurement using the *Enlight 2100* EIT system	The incidence of Adverse Events (AEs) and Serious AEs (SAEs) associated with impedance measurement
The tolerability of impedance measurement using the *Enlight 2100* EIT system	Patient Visual Analogue Scale scores for questions exploring the tolerability of impedance measurement: Patients will be asked to rate their experience with EIT for: • Overall comfort whilst wearing EIT • Overall comfort on removal of EIT • Overall ease of breathing with EIT **and** Withdrawal rate from the study due to inability to tolerate the trial procedure
The change in global impedance in patients randomised to the SAFEx treatment pathway or COT	The change in EELZ and ΔZ measured pre and post extubation
The reintubation rate	The rate of repeat intubation at 24, 48 and 72 h post-extubation

### Data analysis plan

#### Baseline assessment including demographics

The data will be analysed using a standard statistical package. Demographic and clinical characteristics of the sample will be examined descriptively (mean, standard deviation, percentage) to examine the size of each group and to account for the imbalances between the two groups.

#### Primary feasibility and acceptability data analysis

The feasibility parameters and progression criteria for success are outlined in Table 3 and will inform the design of a subsequent larger multi-centre feasibility trial evaluating our SAFEx protocol. If all or some of the progression criteria are not met, investigators will examine contributing factors to feasibility findings and pursue modifications as needed to the study and its delivery prior to any subsequent larger trial.

**Table 3:** Feasibility parameters

Parameter	Data Collection Methods	Criteria for Success
Recruitment Rate	Number of eligible patients approached, consented, and enrolled in the study (with reasons for declines recorded)	30 patients randomised to the study protocol over 12 months
Safety	The incidence of Adverse Events (AEs) and Serious AEs (SAEs) associated with SAFEx treatment	An absence of any Serious Adverse Events associated with the study protocol
Retention Rates	Number of participants completing the study protocol versus those who do not (reasons recorded)	≥ 80% of recruited participants complete the study protocol
Acceptability	Participant questionnaires assessing acceptability of the study interventions	Mean VAS scores of ≥ 7/10 for acceptability questions
Exploratory Endpoints—Extubation Failure Rate and EIT Lung Changes	Extubation failure rate at 72 h calculated using electronic medical records Mean global lung impedance measurements calculated for each group at critical points in the extubation and weaning protocol	100% capture of extubation failure rate at 72 h No progression criteria for EIT lung changes as this is an exploratory endpoint

The primary objective of trial recruitment rate will be expressed as the mean monthly recruitment rate over the complete trial period. Additional feasibility outcomes will be expressed in the following ways:

- Safety outcomes will be expressed as the incidence of AEs or SAEs associated with SAFEx treatment or EIT measurement.
- Consent rate will be expressed as the number of eligible patients who give informed consent divided by the total number of eligible patients approached.
- Attrition rate will be expressed in the following ways:
  ◦ The withdrawal rate from the study, defined as the number of participants requesting to withdraw from the study due to an inability to tolerate the trial procedures
  ◦ The percentage of participants who complete the weaning protocol without breaching any of the physiological patient safety criteria.
- Retention rate: will be expressed as the number of participants completing the study protocol versus those who do not. Reasons for not completing the study protocol will be recorded.
- Acceptability outcomes will be expressed as follows:
  ◦ Mean patient visual analogue scale scores for questions exploring the acceptability of SAFEx treatment compared with that of standard care.
  ◦ Mean patient visual analogue scale scores for questions exploring the acceptability of EIT measurement.
- Data capture outcomes will be expressed in the following ways:
  ◦ The ability to capture the extubation failure rate of patients at 72 h using electronic medical records.
  ◦ The ability to capture and calculate the mean global lung impedance measurements for each group at critical points in the extubation and weaning protocol.

#### Secondary outcome data analysis

The average fraction of inspired oxygen and oxygen flow rate administered in each group before desaturation occurred.

#### Exploratory outcome data analysis

- The change in global impedance between the two groups will be summarised using descriptive statistics. This will include presenting the median values in each group (with interquartile ranges) without conducting hypothesis testing.
- The extubation failure rate: defined as the percentage of patients in each group that require reintubation within 72 h of their planned extubation.

### End of trial

The study will end when the Chief Investigator and Sponsor agree that one or more of the following situations apply:

i. The planned sample size has been achieved
ii. There is insufficient funding to support further recruitment, and no reasonable prospect of additional support being obtained
iii. New information makes it inappropriate to continue to randomise patients to one or more arms of the trial
iv. Recruitment is so poor that completion of the trial cannot be reasonably anticipated

### Ethics and dissemination

#### Safety reporting

Any AEs and SAEs that are identified during trial visits will be assessed, recorded and reported in accordance with the requirements of the trial sponsor.

#### Patient confidentiality

A written case report form (CRF) will be used to collect the study data. Patients will be anonymised immediately after their recruitment for this study. As such, all study data will be recorded on the CRF using the anonymised Unique Code Number only. Anonymised, non-identifiable EIT plethysmographs will be analysed in conjunction with an expert authority in EIT (Dr. Glasiele Cristina Alcala, University of São Paulo Medical School, São Paulo, Brazil).

#### Dissemination

The data from this study will be presented at critical care meetings and published in peer-reviewed critical care journals as well as potentially informing guidelines on the commencement of high-flow nasal oxygen therapy.

#### Trial management

A Trial Management Group consisting of the chief investigator, clinical research fellow and administrative assistant will oversee the running of the trial and meet monthly.

#### Trial status

Recruitment commenced in September 2023 and ended in September 2024. The study is now closed to further patient enrolment. Data analysis is ongoing.

## Discussion

This paper describes a protocol for a randomised feasibility study with novel features in its design. To the knowledge of the authors, this is the first study to consider the impact of HFNT applied prior to extubation on the outcome of extubation and to utilise electrical impedance tomography to measure the effect of the oxygen delivery device on lung aeration peri-extubation.

Practical issues related to the delivery of this study include those shared with all feasibility studies [21–23]. These include eligibility, consent, recruitment, randomisation and the acceptability of the trial specific procedures to patients. Additionally, this study will raise issues with EIT data quality including measurement, processing and analysis. Factors related to critical care research include the feasibility of consent/assent in mechanically ventilated adults who lack capacity and in the delivery of trial specific procedures at extubation. These issues justify the need for a feasibility study and have informed the study objectives.

The strengths of this protocol include the optimal use of HFNT at 60 L/min simultaneously with extubation. This is important because the proposed therapeutic mechanisms of HFNT, such as the generation of PEEP, are flow dependent [7]. In addition, most of the critical care research on extubation failure to date has considered heterogeneous patient populations. In contrast, this study will focus on patients intubated and ventilated with acute respiratory failure, a known subgroup of critical care patients at high risk of extubation failure who have the potential to derive significant benefit from optimal respiratory support post-extubation [24].

There are also limitations to this study. Firstly, only a maximum of 30 patients over 12 months will be recruited, and these patients will be recruited from a single hospital site—the Queen Elizabeth University Hospital, Glasgow, UK. This is less than the number of patients recommended to plan a definitive randomised control trial across multiple sites [20]. This results from a lack of resources to carry out this initial pilot—such as recruitment being facilitated by a single staff member working part-time, having access to only a single Enlight 2100 electrical impedance tomography system and an unknown prevalence of eligible patients. Future multi-centre studies based on this protocol would require additional staff and resources (including HFNT and electrical impedance tomography systems together with their consumables). The decision to extubate will be made by the treating clinician and thus it is not possible to control for the multitude of pre-extubation factors that can influence extubation outcomes, such as the nature of the ventilation and sedation weaning, the degree of patient optimisation pre-extubation and the criteria used to determine extubation readiness [25].

Should the progression criteria for this study be fulfilled, the findings from this feasibility study will initially help plan a multi-centre randomised feasibility trial with an optimal trial design. This would be the next step to then allow statistical powering for a definitive multi-centre randomised controlled trial.

Extubation failure remains a significant burden for critical care services and has major implications for patients, their families and healthcare delivery. There is a paucity of evidence addressing the impact of the flow rate and timing of oxygen delivery at extubation on preventing extubation failure. This study aims to take the first step in addressing this important question.

## Data Availability

After study completion, all anonymised data (including raw and processed EIT data) will be stored in the Enlighten Data Repository (https://researchdata.gla.ac.uk/) for 10 years.

## Abbreviations

AEs: Adverse events
bpm: Beats per minute
COT: Conventional oxygen therapy
CRF: Case report form
EELZ: End-expiratory lung impedance
EIT: Electrical impedance tomography
FiO_2_: Fractional of inspired oxygen
GCS: Glasgow Coma Scale
[H^+^]: Hydrogen ion concentration
HFNT: High-flow nasal therapy
ICU: Intensive care until
MRC: Medical Research Council
NYHA: New York Heart Association
PaCO_2_: Partial pressure of carbon dioxide in arterial blood
PEEP: Positive end-expiratory pressure
SAEs: Serious adverse events
SAFEx: Simultaneous application of flow at extubation
ΔZ: Delta impedance
